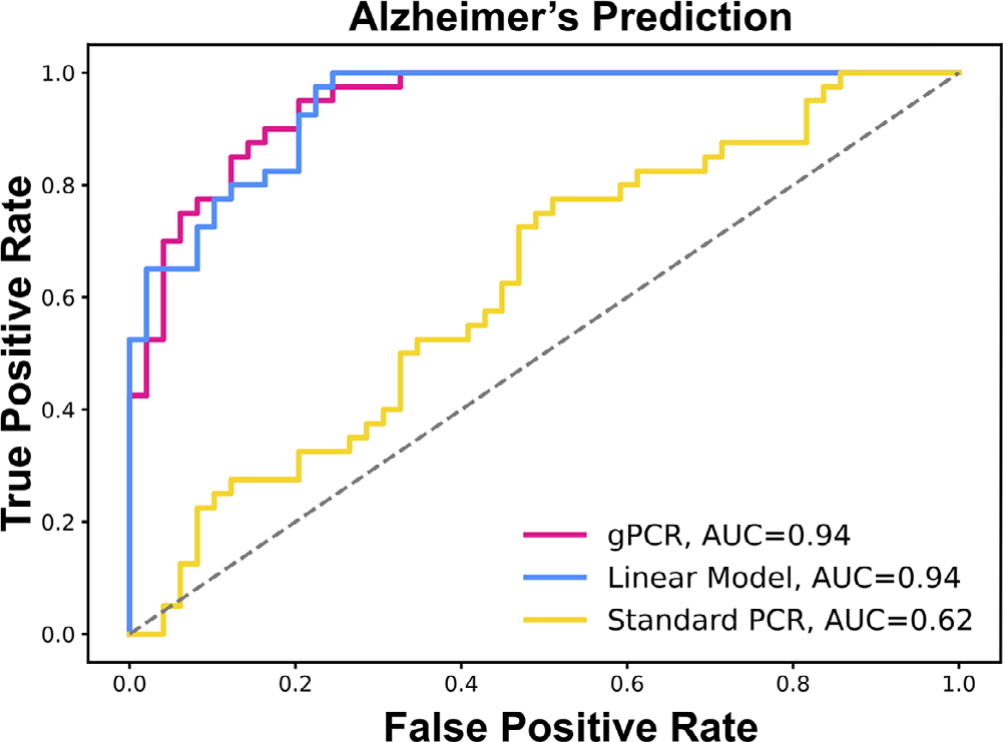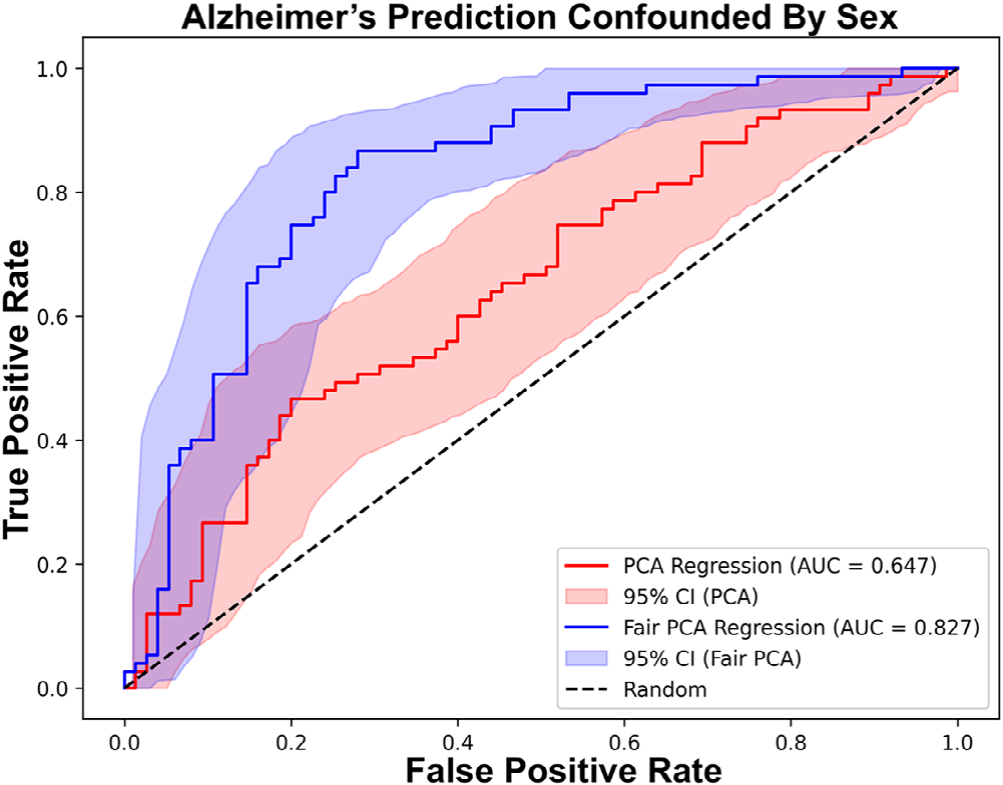# Integrated Fair Machine Learning for Proteomic and Genomic Analysis Workflows

**DOI:** 10.1002/alz70855_100509

**Published:** 2025-12-23

**Authors:** Austin Talbot, Cristina E Trevino, Nicholas T Seyfried, Eric B. Dammer, Erik C.B. Johnson, James J. Lah, Aliza P. Wingo, Thomas S. Wingo, Farhad Imam, Allan I. Levey, Alex Kotlar

**Affiliations:** ^1^ Emory University, Atlanta, GA, USA; ^2^ revXon, Boston, MA, USA; ^3^ Pillar Biosciences, Natick, MA, USA; ^4^ Emory University School of Medicine, Atlanta, GA, USA; ^5^ Goizueta Alzheimer's Disease Research Center, Emory University, Atlanta, GA, USA; ^6^ University of California, Davis, Sacramento, CA, USA; ^7^ Gates Ventures, Seattle, WA, USA

## Abstract

**Background:**

With the growing availability of large genetic and proteomic datasets there is unprecedented need for simplified, scalable methods for integrated omics analysis of Alzheimer's disease (AD).

We have built 8 distinct genomic and proteomic analysis tools for AD community use in the Global Research and Imaging Platform (GRIP), which improve accuracy and interpretability in diverse cases. Included are methods for network discovery, removing the influence of confounders, ancestry inference, variant annotation, natural language filtering, ancestry‐corrected polygenic risk scores, and data harmonization. Here we highlight two methods: generative PCR (gPCR) for detecting phenotypically relevant networks, and FairPCA for removing the influence of confounders.

**Method:**

Latent variable models struggle in ensuring that the latent space is relevant to low‐variance phenotypes. We developed gPCR, which improves on supervised variational autoencoders (SVAEs) to generate phenotypically relevant latent components. We demonstrate its performance by learning a proteomic network associated with AD in a balanced cohort of 300 samples with proteomic data from SomaScan and Tandem Mass Tag mass spectrometry (TMT‐MS). For evaluation, we used a 70/30 train/test split.

To deal with the corresponding problem of removing confounder effects we created FairPCA, an adversarial learning algorithm that ensures principal components are orthogonal to the undesired confounders and utilizes randomization to scale to large datasets. We evaluate FairPCA by removing the influence of sex from protein isoform abundance in 300 TMT‐MS samples, and in removing confounders in a simulated genome wide association study.

**Result:**

gPCR had better predictive performance (AUC=0.94) than traditional PCR (AUC=0.62) and comparable to elastic net regression (AUC=0.94) while also creating relevant protein networks. FairPCA trained to remove the influence of sex outperformed traditional PCA in predicting AD status (FairPCA AUC=0.83, PCA AUC=0.64), while in GWAS simulations the method improved cosine similarity between the inferred betas and the unconfounded true effects (before=0.71, after=.99).

**Conclusion:**

gPCR maintains the predictive power of regularized regression while improving on the generative abilities of SVAEs, and our FairPCA solution simplifies the removal of confounding factors. These and the other 6 tools we have developed for the AD community and made available through GRIP will dramatically accelerate scientific discovery.